# Supplementary material for: Single-cell profiling guided combinatorial immunotherapy for fast-evolving CDK4/6 inhibitor-resistant HER2-positive breast cancer
Source: Nat Commun. 2019 Aug 23;10:3817. doi: 10.1038/s41467-019-11729-1 (PMC6707314; doi:10.1038/s41467-019-11729-1)
Supplement: Supplementary file 1 — Supplementary Information [file 41467_2019_11729_MOESM1_ESM.pdf]

Supplementary Information

Single-cell profiling guided combinatorial immunotherapy for fast-evolving CDK4/6 inhibitor resistant HER2-positive breast cancer

Qingfei Wang[1, 2], Ian H. Guldner[1, 2], Samantha M. Golomb[1, 2], Longhua Sun[1, 2], Jack A. Harris[1, 2], Xin Lu[1, 2, 3] and Siyuan Zhang[1, 2, 3]

[1] Department of Biological Sciences, College of Science, University of Notre Dame, Notre Dame, IN 46556, USA; [2] Mike and Josie Harper Cancer Research Institute, University of Notre Dame, South Bend, IN 46617, USA; [3] The Indiana University Melvin and Bren Simon Cancer Center, Indianapolis, IN 46202, USA

Correspondence and requests for materials should be addressed to Q.W. (email: qwang9@nd.edu) or to S.Z. (email: szhang8@nd.edu)

Supplementary Figure 1

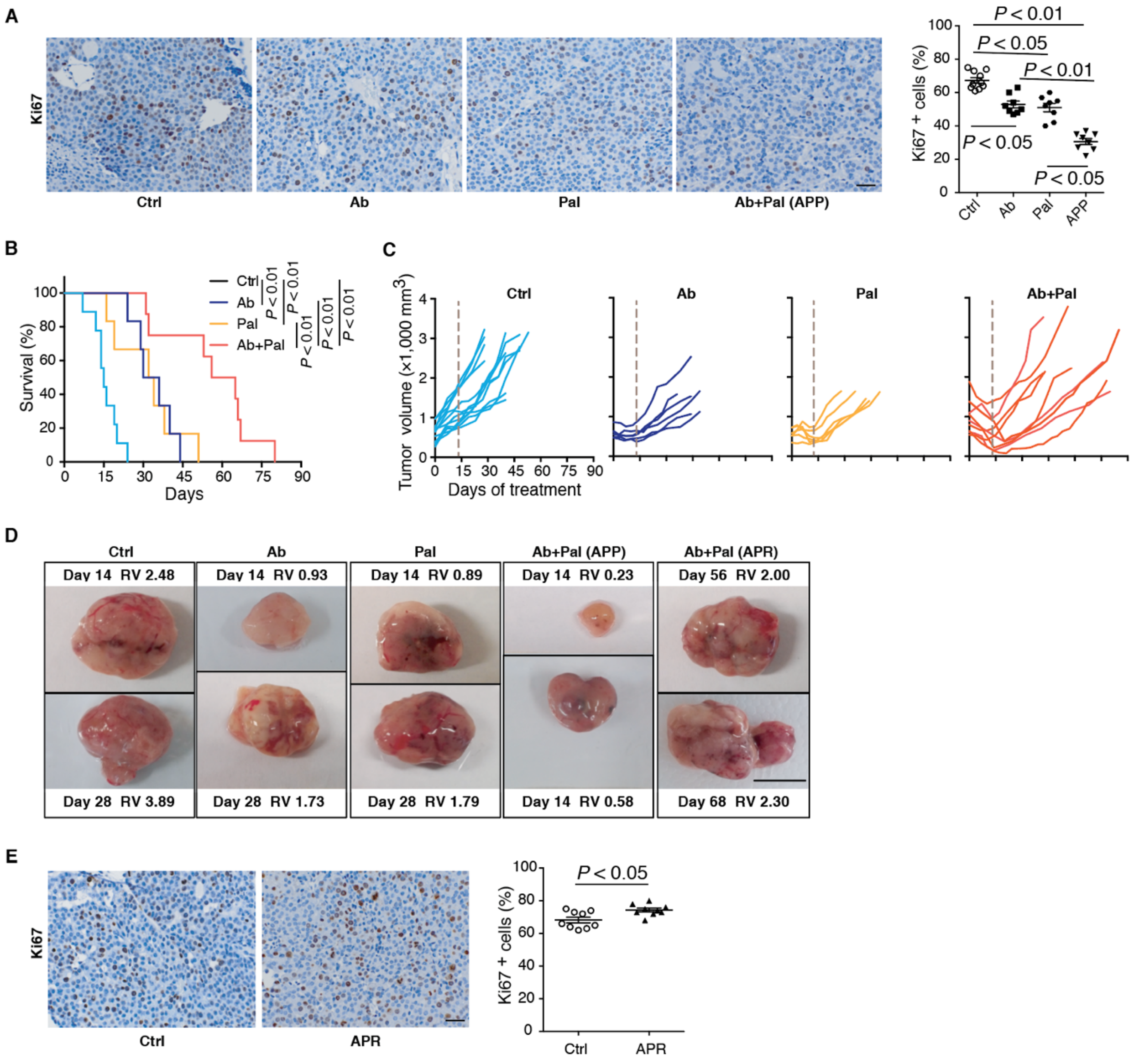

Supplementary Figure 1. Response and resistance to anti-Her2/Neu antibody plus CDK4/6 inhibitor Palbociclib combination in spontaneous Her2/Neu-positive breast cancer. (**A**) Representative images and quantification of Ki67 immunohistochemistry staining. MMTV-neu202^{Mul} mice (bearing tumor > 500 mm^3) were treated with Ctrl, Ab or Pal alone, or Ab+Pal for 14 days, then tumors were harvested. Ctrl, vehicle treated control; Ab, anti-Her2/Neu antibody; Pal, CDK4/6 inhibitor Palbociclib. Scale bar, 20 μm. *P*-value by one-way ANOVA with Tukey's test. (**B**) Survival time to doubled tumor volume (n=9,6,6 and 8 for Ctrl, Ab, Pal and Ab+Pal). *P*-value by log-rank (Mantel-Cox) test. (**C**) Individual tumor growth kinetics with Ctrl (n=12), Ab (n=6) or Pal (n=5) alone, or Ab+Pal (n=10) treatment. Dash line indicating 14-day's treatment. (**D**) Representative images of tumors with indicated treatment. RV: relative tumor volume. RV = tumor volume at the indicated time (days of treatment) / volume at the start point of treatment. Scale bar, 1cm. (**E**) Representative images and quantification of Ki67 immunohistochemistry staining on tumors derived from mice with 7 weeks' treatment. Scale bar, 20 μm. *P*-value by Student's t-test. Error bars in **A** and **E** represent SEM.

Supplementary Figure 2

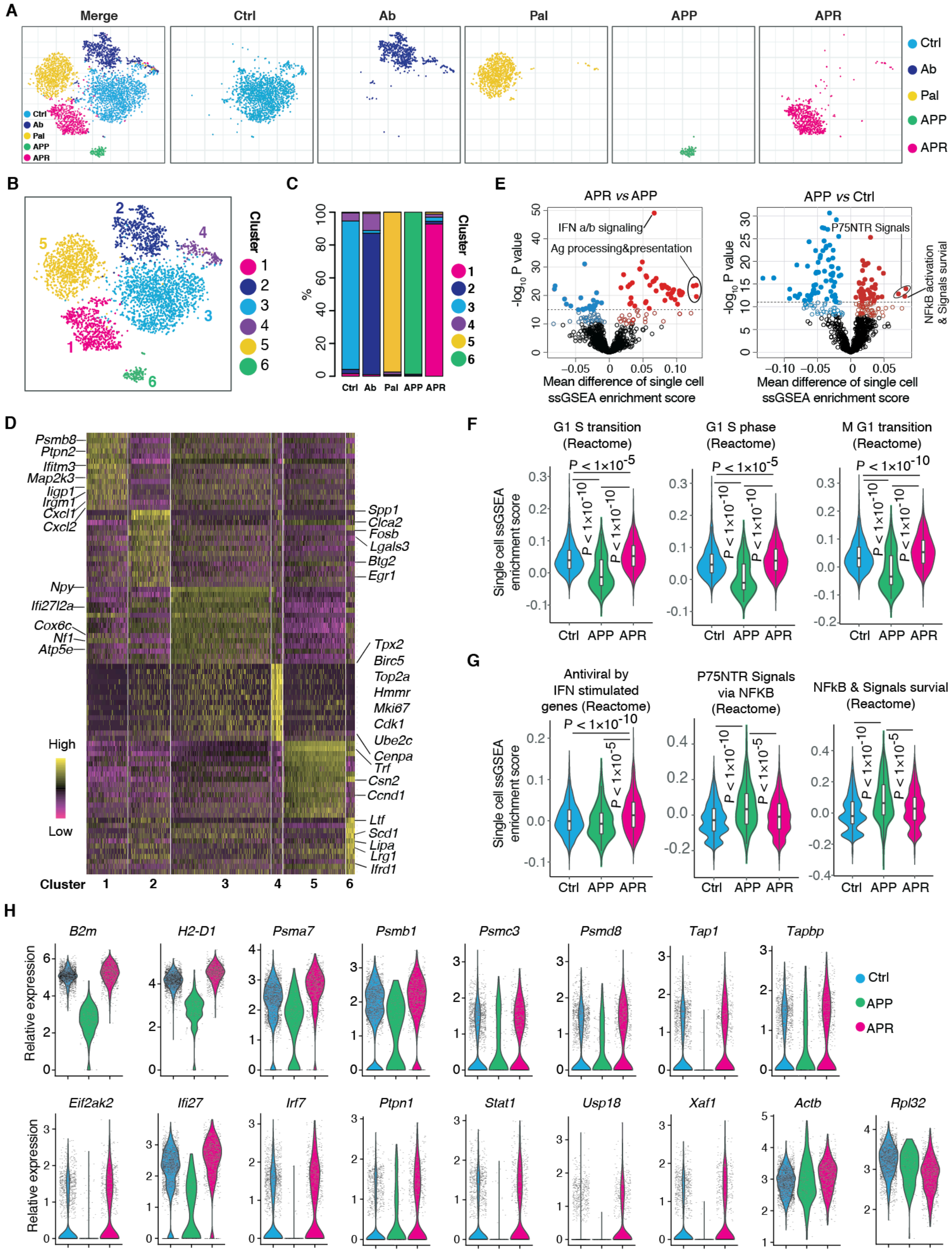

Supplementary Figure 2. Single-cell RNA sequencing of tumor cells from responsive and resistant tumors to anti-Her2/Neu antibody plus CDK4/6 inhibitor Palbociclib combination treatment. (**A**) Clustering of 4,711 tumor cells by single-cell expression profiles and t-distributed stochastic neighbor embedding (t-SNE) plots colored by different treatment [merged (left) or subdivided into individual treatment group (right)] are shown. Each point represents a single cell. (**B**) t-SNE plot from (**A**) is colored by cluster. (**C**) Abundance of each cluster [as clustered in (**B**)] in tumors with indicated treatment. (**D**) Heatmap of top differentially expressed (marker) genes of each cluster. Each column represents a single cell. Cluster 1 is predominantly composed of cells from APR tumors with high expression levels of *Psmb8, Ptpn2, Ifitm3, Map2k3, Iigp1, Irgm1, Cxcl1* and *Cxcl2*. Cluster 6 mostly represented the cells from APP tumors with high expression of *Ltf, Scd1, Lipa, Lrg1 and Ifrd1* genes. (**E**) Volcano plots comparing ssGSEA enrichment score for 1053 canonical pathways/gene sets of the C2 collection of Molecular Signatures Database between APR and APP (left), APP and Ctrl (right), based on scRNA-Seq data. Each point represents one pathway/gene set. X-axis, mean difference of single-cell ssGSEA enrichment score; Y-axis, -log10 (*P*-value by t-test). (**F** and **G**) ssGSEA enrichment score violin plots for single cells from each treatment group for cell cycle related signatures (**F**) and for 'antiviral by IFN stimulated genes', death receptor 'P75 NTR signaling' and 'NFκB is activated and signals survival' signatures (**G**). *P*-value by Student's t-test (two-tailed). (**H**) Expression of genes involved in antigen processing and presentation (upper panel), IFN signaling and response (lower panel) in individual cells from tumors with indicated treatment. Upper panel, genes encoding mouse major histocompatibility complex (MHC) class I molecules (*B2m* and *H2d1*), peptide transporters (*Tap1*), proteasome family members for protein degradation and peptide production (e.g. *Psma7, Pamb1, Psmc3* and *Psmd8*) and transporter-MHC interactions (*Tapbp*) exhibited either higher expression levels or more expressing cells in APR tumors. Lower panel, genes involved in innate immune response to viral infection (*Eif2ak2*, double-stranded RNA-activated protein kinase), regulation of interferon signaling (*Ptpn1*), interferon-responsive transcription factors (*Irf7* and *Stat1*) and interferon stimulated/ inducible genes (*Ifi27, Usp18, Xaf1*) were increased in APR tumors. Each point represents a single cell. Expression of housekeeping genes *Actb* and *Rpl32* in individual cells are also shown.

Supplementary Figure 3

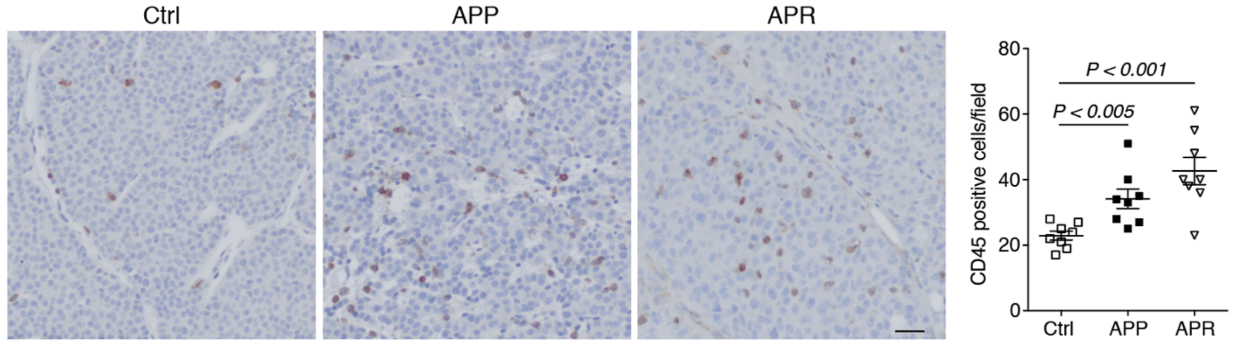

Supplementary Figure 3. Ab+Pal treatment increased tumor infiltrating leukocytes. Representative images and quantification of CD45 immunohistochemistry staining for Ctrl, APP and APR tumors. Scale bar, 20 μm. Error bars represent SEM. *P*-value by one-way ANOVA with Tukey's test.

Supplementary Figure 4

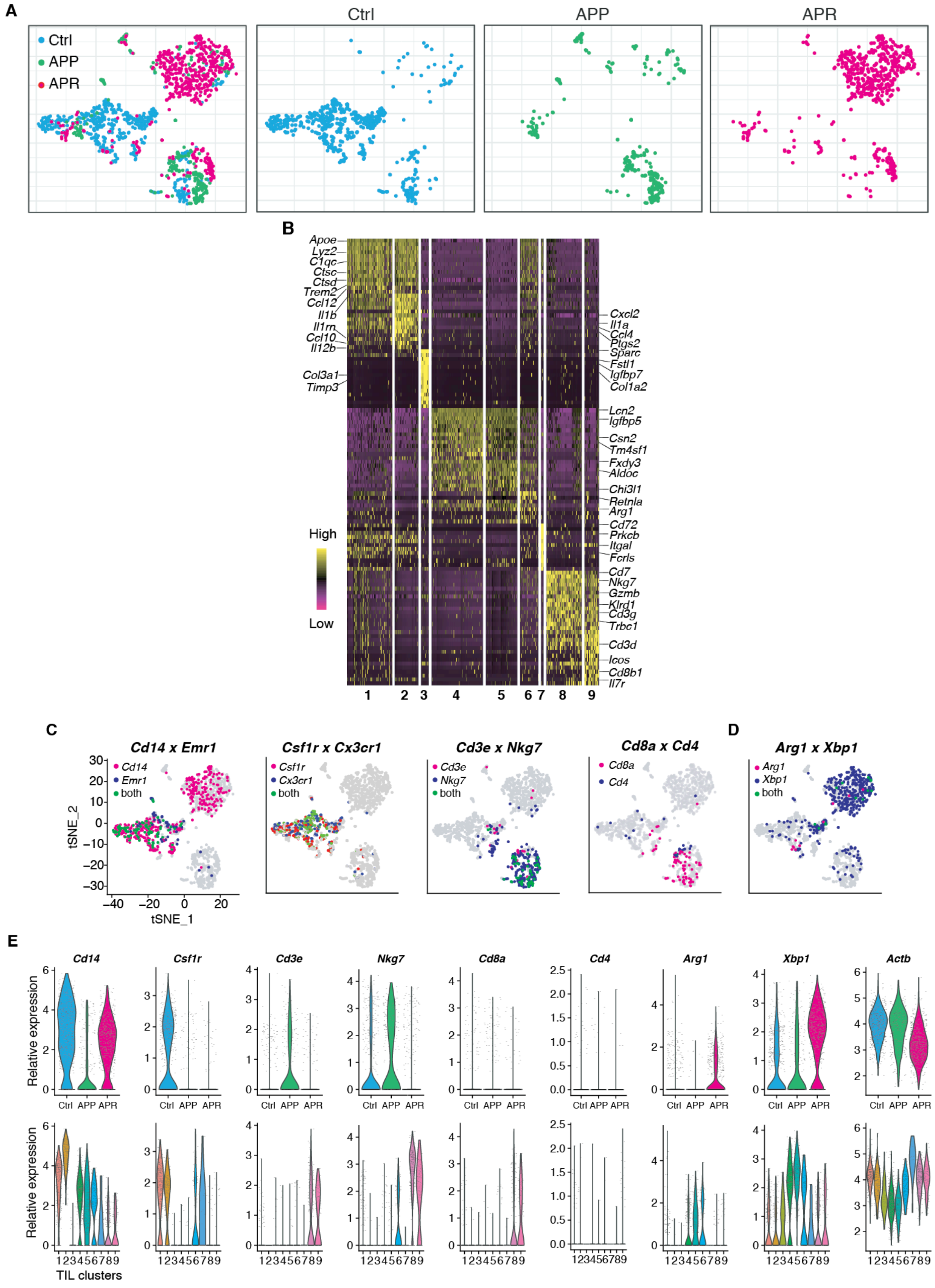

Supplementary Figure 4. Single-cell RNA sequencing of tumor infiltrated leukocytes derived from Ab+Pal treatment responsive and resistant tumors. (**A**) Clustering of 1,444 TILs derived from Ctrl, APP and APR tumors and t-SNE plots colored by different phenotypes [merged (left) or subdivided into individual group (right)] are shown]. Each point represents a single cell. (**B**) Heatmap of top differentially expressed or marker genes of each cluster from experiment (**A**). Each column represents a single cell. Cluster 1 (281 cells) and cluster 2 (147 cells) showed marker genes of macrophage (e.g. Apoe, Lyz2, C1qc). Cluster 8 (222 cells) and Cluster 9 (92 cells) showed high expression of NK and/or T-cell genes (e.g. *Nkg7, Gramb, Cd3g, Cd3d, Trbc2, Cd8b1*). Cluster 3 [50 cells, mostly (38/50) derived from APP] was characterized by *Sparc, Fstl1, Igfbp7, Timp3* and collagen genes, including *Col1a1, Col1a2* and *Col3a1*. Cluster 7 (17 cells all from APP) comprised genes characteristic of both dendritic cell and macrophage, such as *Cd72, Itgal* and *Fcrl5*. Clusters 4 (327 cells) and 5 (191 cells) displayed high expression of monocyte genes (e.g. *Lcn2, Csn2* and *Chi3l1*). Cluster 6 (117 cells) showed intermediate expression of both clusters 1&2 and clusters 4&5-related genes. (**C** and **D**) Expression of key marker genes used for immune cell-type identification and annotation was overlaid on t-SNE plots. (**E**) Expression distribution of key marker genes [as mentioned in (**C** and **D**)] and housekeeping gene *Actb* among tumors with different phenotypes (upper) and different TIL-clusters (lower) were shown. Each dot represents a single cell.

Supplementary Figure 5

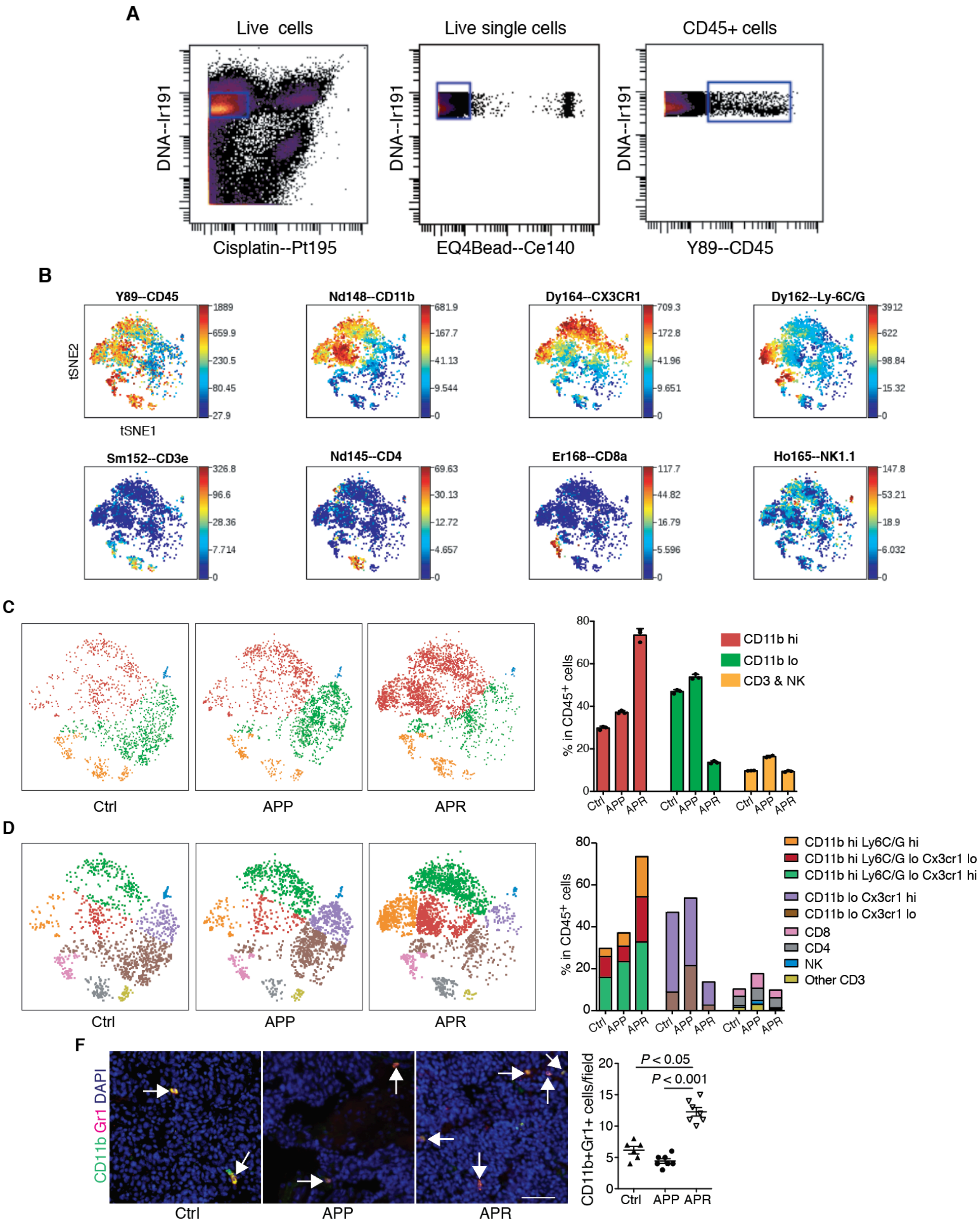

Supplementary Figure 5. Characterization of infiltrating immune cell populations in responsive and resistant tumors to Ab+Pal treatment. (**A**) Preliminary gating for mass cytometry samples. Iridium-191 and cisplatin-195 were used to select live single cells and subsequently CD45+ cells were selected for further analysis. (**B**) Representative plots showing the expression of markers used for cell characterization/gating overlaid on viSNE plots. viSNE plots were generated by Cytobank, based on the t-SNE algorithm. Each point represents a single cell and the color gradient represents marker's intensity (expression level). (**C** and **D**) Classification of CD45+ TILs populations on viSNE plots and quantification. (**F**) Representative images and quantification of CD11b and Gr1 immunofluorescence staining for Ctrl, APP and APR tumors. Arrows indicate CD11b and Gr1 double positive cells. Scale bar, 50 µm. Error bars represent SEM. *P*-value by one-way ANOVA with Tukey's test.

Supplementary Figure 6

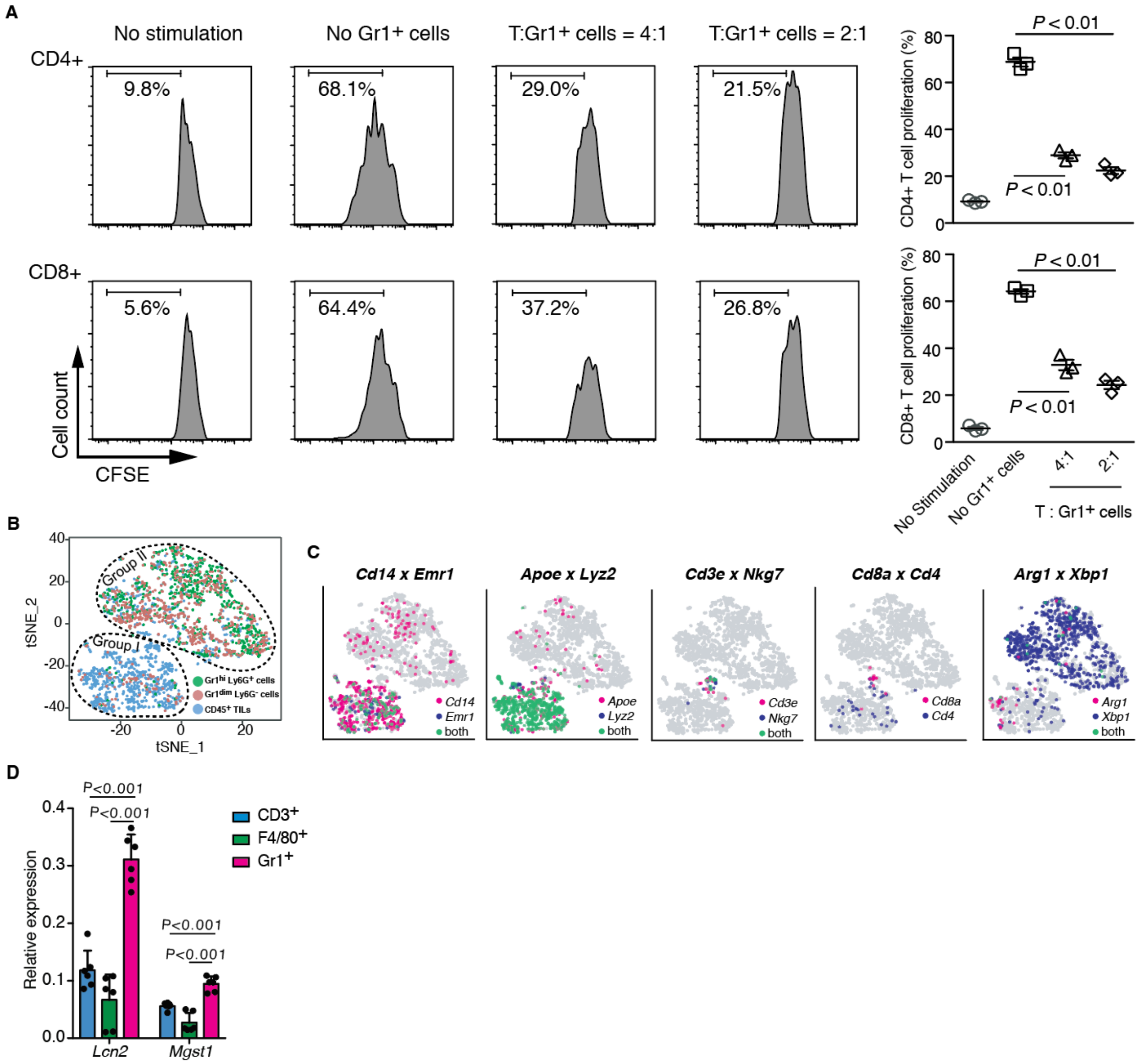

Supplementary Figure 6. Functional characterization and single-cell RNA sequencing of tumor infiltrated MDSCs. (**A**) Gr1+ MDSCs isolated from Ab+Pal treatment resistant tumors inhibited CD3/CD28 stimulated proliferation of CD4+ and CD8+ T cells *in vitro*. Left, representative flow cytometry histograms measuring carboxyfluorescein succinimidyl ester (CFSE); right, quantification of T-cell proliferation. Error bars represent SEM. *P*-value by Student's t-test. (**B**) t-SNE plot of scRNA-seq data from $CD45^+$ TILs, $Gr1^{high}$ $Ly6G^+$ cells and $Gr1^{dim}$ $Ly6G^-$ cells. Each point represents a single cell. (**C**) Expression of key marker genes used for immune cell-type identification and annotation was overlaid on t-SNE plots as shown in (**B**). Expression of macrophage genes (*CD14, Emr1 or F4/80, Apoe* and *Lyz2*), T/NK-cell related genes (*Cd3e, Nkg7, Cd4 and Cd8a*), *Arg1* and *Xbp1* were shown. (**D**) mRNA levels of *Lcn2* and *Mgst1* in sorted T cells ($CD3^+$), macrophages ($F4/80^+$) and MDSCs/IMCs ($Gr1^+$) from APR tumors were quantified by RT-PCR. Error bars represent SD. *P*-value by one-way ANOVA with Tukey's test.

Supplementary Figure 7

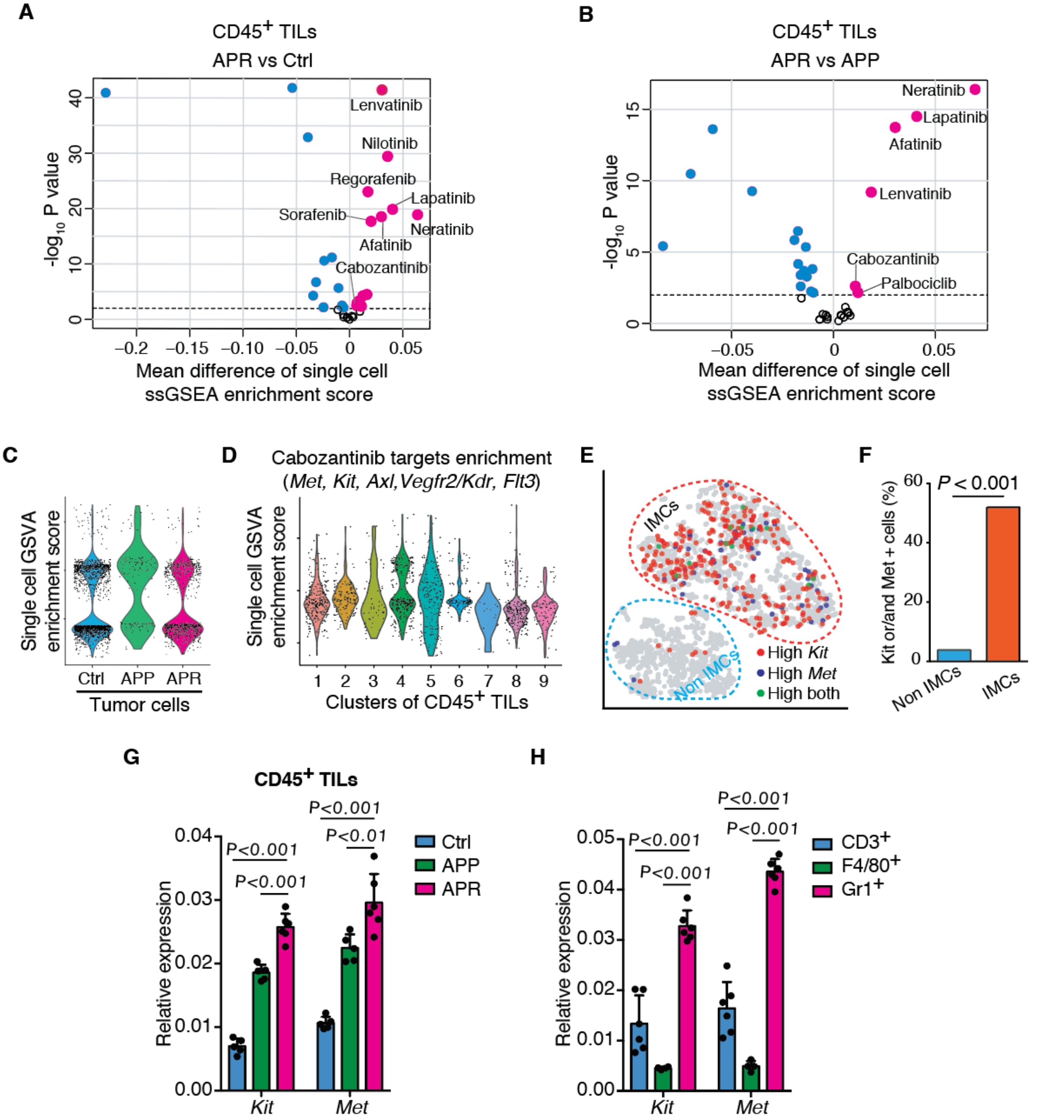

Supplementary Figure 7. Identification of cabozantinib as a potential IMCs-targeted drug by single-cell RNA-seq analysis. (**A** and **B**) Volcano plots comparing enrichment score for drug target genes of FDA approved protein kinase inhibitors between TILs from APR and Ctrl tumors (**A**), and from APR and APP tumors (**B**). Each point represents an inhibitor. X-axis, mean difference of ssGSEA enrichment score between groups; Y-axis, -log10 (*P*-value by t-test). (**C**) Enrichment analysis of cabozantinib target genes across tumor cells grouped and plotted by different phenotypes. Each point represents a single cell. (**D**) Distribution of enrichment score for cabozantinib target genes among TIL-clusters as identified in Fig.2A. (**E**) Distribution of *Kit* and/or *Met* expression cells on t-SNE of scRNA-seq data for CD45^{+} TILs and Gr1^{+} cells in experiment of **Fig.2 g**. Each point represents a single cell. (**F**) Abundance of Kit and/or Met expressing cells in IMCs and non-IMCs as annotated in (**E**). *P*-value by Chi-square test. (**G** and **H**) mRNA levels of *Kit* and *Met* in CD45^{+} TILs form Ctrl, APP and APR tumors (**G**) and in sorted T cells (CD3^{+}), macrophages (F4/80^{+}) and MDSCs/IMCs (Gr^{+}) from APR tumors (**H**) were quantified by RT-PCR. Error bars represent SD. *P*-value by one-way ANOVA with Tukey's test.

Supplementary Figure 8

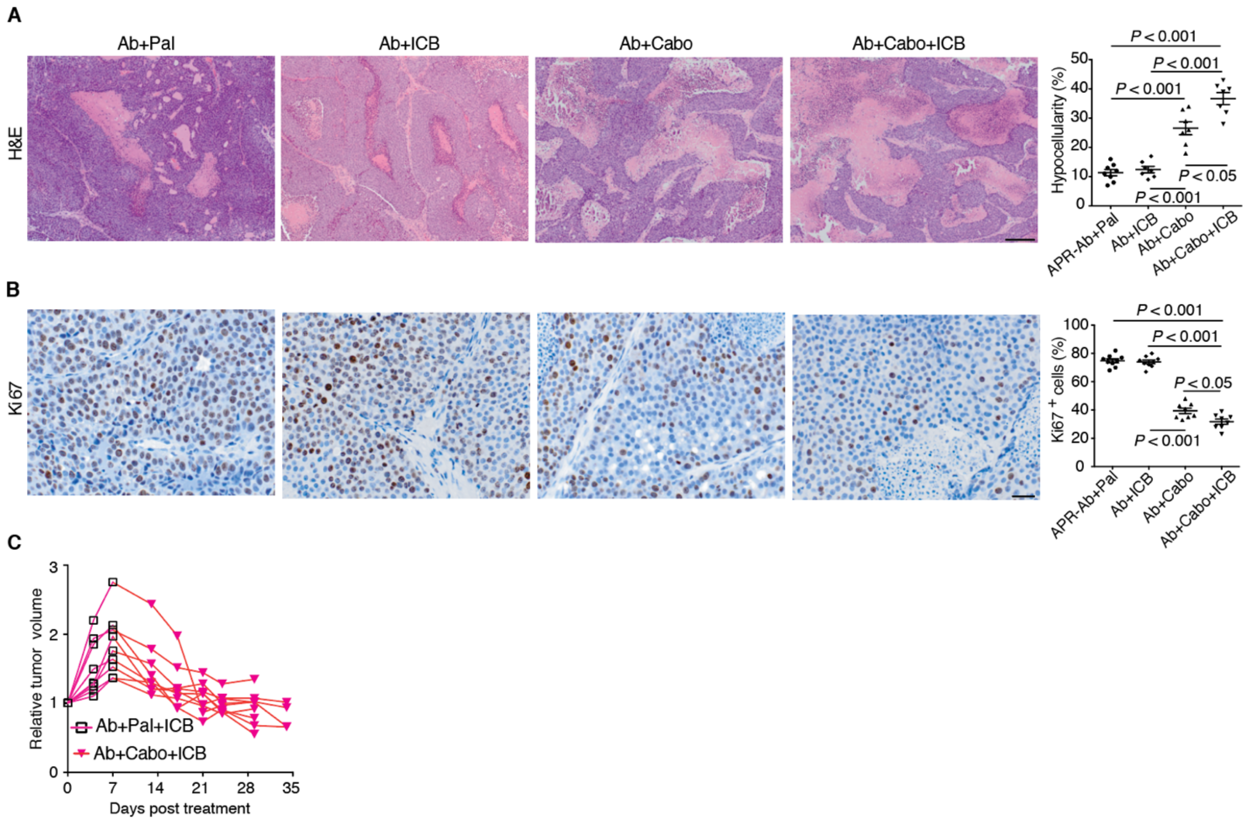

Supplementary Figure 8. Therapeutic activity of Ab+Cabo or Ab+Cabo+ICB against Ab+Pal resistant tumors. (**A**) Representative images of H&E staining for tumors with indicated treatment and quantification of hypocellularity. Scale bar, 100 µm. (**B**) Representative images and quantification of Ki67 immunohistochemistry staining tumors with indicated treatment. Scale bar, 20 µm. (**C**) Relative volumes of Ab+Pal resistant tumors after sequential treatment with Ab+Pal+ICB and Ab+Cabo+ICB (as in Fig.3F). Ab+Pal resistant tumors were first treated with Ab+Pal+ICB for 1 week then switched to Ab+Cabo+ICB treatment for 3 weeks. Cabo, protein kinase inhibitor cabozantinib; ICB, immune checkpoint blockades, cocktail of anti-CTLA4 and anti-PD-1 antibodies. Error bars represent SEM. *P*-value by one-way ANOVA with Tukey's test.

Supplementary Figure 9

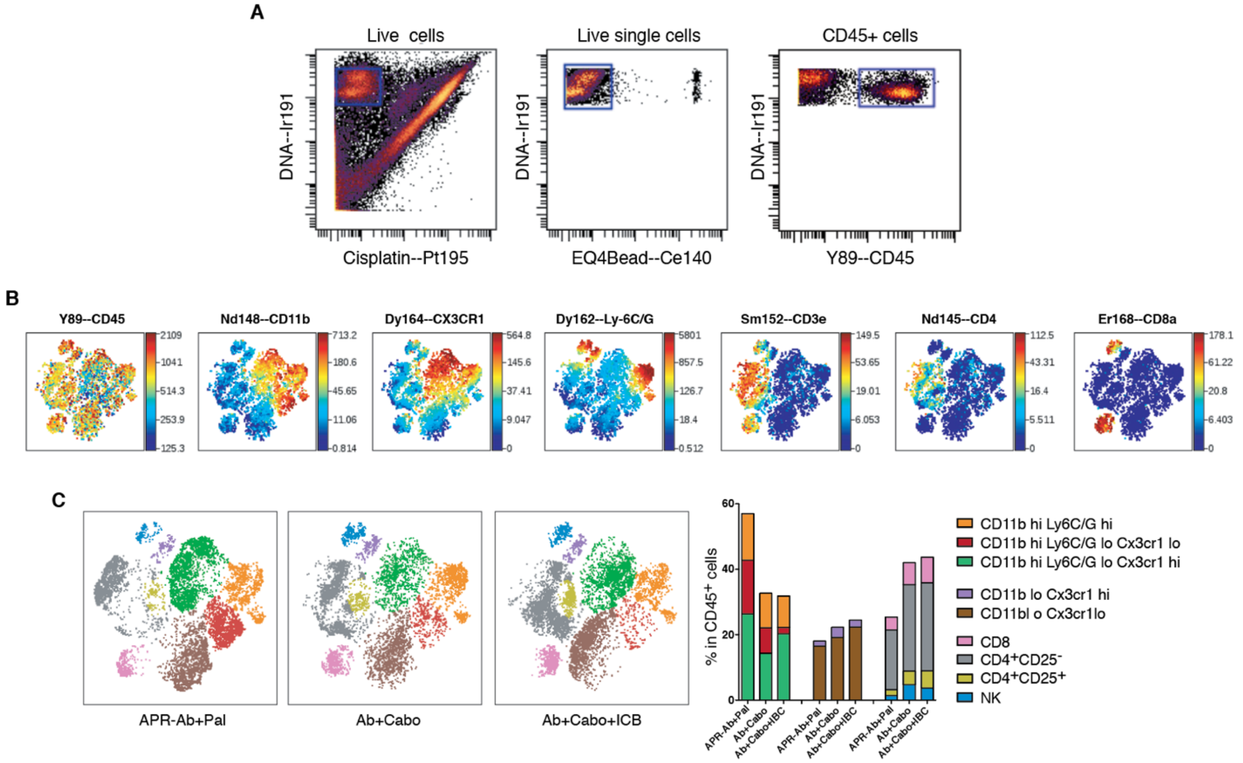

Supplementary Figure 9. Characterization of infiltrating immune cell populations in tumors after Cabo and ICB treatment. (**A**) Preliminary gating for mass cytometry samples after Cabo and ICB treatment. Iridium-191 and cisplatin-195 were used to select live single cells and subsequently CD45$^+$ cells were selected for further analysis. (**B**) Representative plots showing the expression of markers used for cell characterization/gating overlaid on viSNE plots. viSNE plots were generated by Cytobank, based on the t-SNE algorithm. Each point represents a single cell and the color gradient represents marker's intensity (expression level). (**C**) Classification of CD45$^+$ tumor infiltrated immune cell populations on viSNE plots (left) and quantification (right).

Supplementary Figure 10

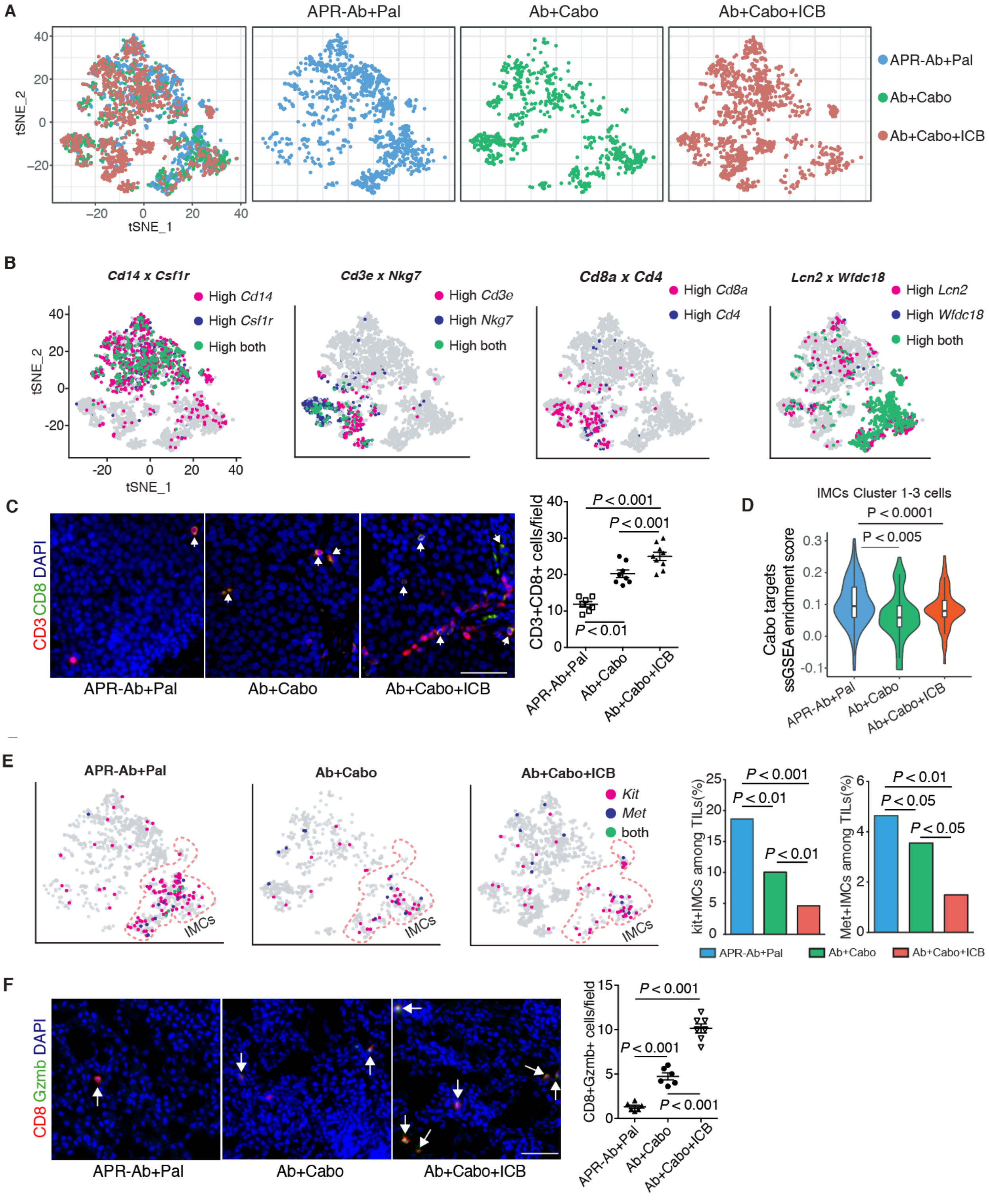

Supplementary Figure 10. **Single-cell RNA sequencing of tumor infiltrated leukocytes after Cabo and ICB treatment.** (**A**) t-SNE plots of 3,168 TILs derived from APR tumors with continuous Ab+Pal, Ab+Cabo or Ab+Cabo+ICB treatment, merged (left) or subdivided into individual group (right) were shown. Each point represents a single cell. (**B**) Expression of key marker genes used for immune celltype identification and annotation was overlaid on t-SNE plots. (**C**) Representative images and quantification of CD3 and CD8 immunofluorescence staining. Arrows indicate CD3 and CD8 double positive cells. Scale bar, 50 μm. Error bars represent SEM. *P*-value by one-way ANOVA with Tukey's test. (**D**) Enrichment scores of cabozantinib target genes among tumor infiltrated IMCs after indicated treatment. *P*-value by Student's t-test. (**E**) Distribution of Kit and/or Met expression cells on t-SNE plot of scRNA-seq data from (**A**), and abundance of *Kit* or *Met* expressing IMCs within $CD45^+$ TILs after indicated treatment. *P*-value by three-sample Chi-square test. (**F**) Representative images and quantification of CD8 and Granzyme B immunofluorescence staining. Arrows indicate CD8 and Granzyme B double positive cells. Scale bar, 50 μm. Error bars represent SEM. *P*-value by one-way ANOVA with Tukey's test.

Supplementary Table 1

Supplementary Table 1. Gr-1^{+} MDSCs signature (Top 300 differentially expressed genes of MDSCs clusters by scRNA-seq as shown in Fig. 2h)

1--60	61--120	121--180	181-240	241--300
Lcn2	Cp	Sorbs2	Perp	Arhgap29
Wfdc18	mt-Nd1	Atp6v1c2	Shf	Lyrm5
Trf	Mfge8	Kcnk1	St8sia6	Fuom
Aldoc	Ano1	Tesc	Dhrs7	Plcb4
Epcam	Duoxa1	Chn2	Tmc4	Phactr1
Col9a1	Tc2n	Sox10	Echdc2	Lsr
Aqp5	Fnbp1l	Mrps6	Slc29a1	Cnbp
Apoc1	Clca2	Atp5g3	Rps3a1	Abhd2
Mgst1	Emid1	1110008P14Rik	Ceacam10	Gstm5
Fxyd3	Fkbp11	Mpzl1	Lgalsl	Ndufb11
Rbp1	BC006965	Car6	Map1lc3a	Gnb2l1
Fermt2	Wbp5	Pdzrn3	Comp	Myo10
Krt18	Sfrp1	Secisbp2l	Bcas2	Klhdc2
Atp1b1	Galnt3	Rps5	Abcg2	Smagp
Cald1	Krt8	Ccnd1	Serbp1	Pfdn1
Dbi	Cox6c	Elf5	Golim4	1110001J03Rik
Rps14	Etnk1	Tspan3	Gne	Esf1
Rpl32	Sdc2	Nes	Rbp7	Rhoj
Igfbp5	Pgp	Strbp	Serpinh1	Pradc1
Ehf	Hlf	Me1	Tgfb3	mt-Nd5
Csn3	Psma7	Spry2	Tspan13	Gpr64
Lalba	Egln3	Ceacam1	Scgb2b27	Csnk1a1
Serpine2	Xbp1	Sox4	Pgrmc2	Kitl
Stk39	Cldn10	Rab25	Fam174b	Tmem216
Spint2	Clu	Fam115a	Npdc1	F3
Lmo4	Cystm1	Rab15	Slc35a1	Cd81
Socs2	Rps21	Wwc1	Sox9	Bhlhe40
Col11a1	Ndufb9	Cd99l2	Ptprg	Daam1
Cd9	Cgnl1	Dstn	Sec11c	Krtcap2
Ifi27	Kif21a	Uqcr10	Fkbp4	Irx3
Cldn3	Scd1	Ptprf	S100a16	Marveld3
Actn4	Phlda1	Sntb2	Tuba4a	Il17re
mt-Cytb	Homer2	Cox8a	Ckmt1	Dynll2
Crip2	Bzw2	Prlr	Rpl26	Spcs1
Rpl8	Chchd10	Ndufb2	Pxmp4	2010107E04Rik
4931406C07Rik	Fblim1	H1f0	Eef1b2	Slc16a2
Acsl4	Col9a3	mt-Nd2	Ociad2	Hook1
Sdc4	Fam213a	Mpc2	Rps24	Gabrp
Rpl13a	Mia	Pcdh10	Fam150b	Rhoc
Urah	Ehd3	Tspan1	Tecr	2700089E24Rik
Cpe	Gnas	Cox7a2	Itga8	Atp5f1
Cmas	Cldn7	Ndufa6	Hhipl2	H2afj
Tm4sf1	Mgll	Cd24a	Errfi1	Crispld2
Nedd4	Nop10	Rcn1	Car12	Aldoa
Atp5c1	S100a1	Ssr2	Atp5e	Copz2
Nfib	Dsg2	Ppfibp2	Ppdpf	Ergic2
Chi3l1	Tmbim6	Ndufc1	Col9a2	Wwc2
Akap12	Ppap2b	Irx2	Acat1	Cox6b1
Slc12a2	4930503L19Rik	Tspan8	Fgfr1	Fos
Palmd	Atp5j	Fkbp3	Iqgap2	Emg1
Ncald	Deb1	Tcn2	Mpzl2	Zfp385b
Kcnn4	Npnt	Kit	Acadsb	Dab2ip
Ldhb	Fam3c	Itga6	Cct7	Stox2
1600029D21Rik	Ndufa5	Cnn3	St3gal5	Amotl1
Erbb2	Hey1	Papss1	0610007N19Rik	Mien1
Etv1	Tfap2c	Lbp	Timm8b	Pcdh18
Ndufa4	mt-Nd4	Atp6v1b1	Ndufb5	Mfsd7c
Folr1	Car2	Deptor	Kcnk5	Pnpla8
Lman1	Smim22	Pcbp1	Gata3	Tsc22d1
Rps27l	Tpd52l1	Sdpr	Dad1	Irx1

Supplementary Table 2

Supplementary Table 2. Primer sequences

Gene Symbol	GenBank Accession	Forward Primer (5' to 3')	Reverse Primer (5' to 3')
Lcn2	NM_008491	TGGCCCTGAGTGTCATGTG	CTCTTGTAGCTCATAGATGGTGC
Mgst1	NM_019946	CTCAGGCAGCTCATGGACAAT	GTTATCCTCTGGAATGCGGTC
Kit	NM_001122733	CTCCCCCAACAGTGTATTCAC	TAGCCCGAAATCGCAAATCTT
Met	NM_008591	GTGAACATGAAGTATCAGCTCCC	TGTAGTTTGTGGCTCCGAGAT
Actb	NM_007393	GGCTGTATTCCCCTCCATCG	CCAGTTGGTAACAATGCCATGT

Supplementary Methods

CyTOF antibodies

The following pre-conjugated antibodies purchased from Fluidigm were used in this study: CD45-089Y (3089005B, 30-F11); Ly-6G-141Pr (3141008B, 1A8,); CD11c-142Nd (3142003B, N418); CD45R-144Nd (3144011B, RA3-6B2); CD4-145Nd (3145002B, RM4-5); CD11b-148Nd (3148003B, M1/70); CD44-150Nd (3150018B, IM7); CD25-151Eu (3151007B, 3C7); CD3e-152Sm (3152004B, 145-2C11); PD-L1-153Eu (3153016B, 10F.9G2); CTLA-4-154Sm (3154008B, UC10-4B9); PD-1-159Tb (3159024B, 29F.1A12); Ly-6C-162Dy (3162014B, HK1.4); CX3CR1-164Dy (3164023B, SA011F11); NK1.1-165Ho (3165018B, PK136); c-Kit-166Er (3166004B, 2B8); CD8a-168Er (3168003B, 53-6.7); CD86-172Yb (3172016B, GL1); I-A/I-E-209Bi (3209006B, M5/114.15.2).

T-cell suppression assay

MDSCs were isolated from Ab+Pal treatment resistant tumors by Ficoll separation followed by using a mouse MDSC Isolation Kit (Miltenyi Biotec, 130-094-538). Isolate lymphocytes from spleen of wild-type FVB/N mice were labeled with carboxyfluorescein succinimidyl ester (CFSE) (2 μM) (Invitrogen, C34554), stimulated with CD3/CD28 magnetic beads (Invitrogen, 11452D), and cultured alone or with MDSCs at different ratios for 3 days. Cells were then collected and stained with anti-CD4-PE and anti-CD8a-APC (BioLegend, 100407 & 100711), CFSE intensity was quantified by flow cytometry and T-cell proliferation was analyzed.

Histology and immunohistochemistry

Tumor tissues were fixed in 10% neutral buffered formalin, processed routinely, and embedded in paraffin. H&E staining of paraffin-embedded tumor sections were used to quantify the hypocellular regions. Paraffin-embedded sections (4 μm) were subjected to antigen retrieval in a pressure cooker with sodium citrate buffer (PH=6.0) and incubated with antibodies specific for CD45 (BioLegend, 103103, 1:100), Ki-67 (DAKO, 1:200) overnight at 4°C. Biotin-conjugated secondary antibodies were used. Remaining steps were performed using Vectastain ABC kits (Vector Laboratories). Slides were counterstained with hematoxylin. To quantify the positive staining cells, the numbers of infiltrating $CD3^+$ or $Ki67^+$ cells were counted per field of view after examination of at least 10 fields of each section (200X), and the percentage of positive cells for Ki-67 was evaluated. Images were acquired using a Zeiss microscope with Axiovision software (Carl Zeiss, Inc.).

Immunofluorescence staining

FFPE sections were used for CD3 and CD8 IF staining. Sections (4 μm) were subjected to antigen retrieval in a pressure cooker with sodium citrate buffer (PH=6.0), blocked with R.T.U. Animal Free Blocker and

Diluent (Vector Laboratories) for 1 h and then incubated with antibodies for CD3 (Abcam, ab16669, 1:300), CD8 (Thermo Scientific, 14-0808-82, 1:150) overnight at 4°C, followed by washing with PBS containing 0.05% TWEEN-20. Sections were then incubated with goat anti-Rabbit- Alexa Fluor 488 and goat anti-Rat- Alexa Fluor 594 (Thermo Scientific, A11034 and A11007, 1:500) in the blocking solution for 1 h at room temperature. After washing, sections were stained with DAPI to visualize nuclei.

Cryosections were used for CD11b, Gr1, CD8 and granzyme B IF staining. For preparation of cryosections, dissected tissues were embedded in Tissue-tek O.C.T. (Electron Microscopy Sciences) and frozen on dry ice. Frozen tissues were stored at –80 °C until they were sectioned at 7 μm. For multicolored immunofluorescence staining, O.C.T. tumor cryosections were briefly air dried and fixed with 3% paraformaldehyde at room temperature for 15 min. Sections were then blocked with R.T.U. Animal Free Blocker and Diluent (Vector Laboratories) for 1 h and incubated with primary antibodies for CD11b-Alexa Fluor 488 (BioLegend, 101217, 1:50), Gr1-Alexa Fluor 594 (BioLegend, 108448, 1:50), CD8 (Thermo Scientific 14-0808-82, 1:150), granzyme B-FITC (BioLegend, 372205, 1:50). Secondary antibody goat anti-Rat-Alexa Fluor 594 (Thermo Scientific, A11007, 1:500) was used for CD8 staining. After washing, sections were stained with DAPI to visualize nuclei. Immunofluorescence imaging was performed on a multicolour fluorescent microscope (Leica DM5500 B). Five random fields were acquired from each biological sample for quantification of positive stained cells.

Flow cytometry sorting

Cryopreserved mammary tumors were enzymatically digested followed by density centrifugation. Cell suspensions were incubated with TruStain fcX (BioLegend, 101319) in 100uL MACS buffer (PBS with 0.5% BSA and 2 mM EDTA) for 15 minutes at room temperature. Cells were then incubated with pre-conjugated antibodies including CD45-APC (BioLegend, 103111), CD3-PE (Phycoerythrin) (BioLegend, 100205), F4/80-FITC (Fluorescein Isothiocyanate) (BioLegend, 123107), Gr1-APC/Cy (BioLegend, 108423). Flow cytometry sorting was performed on BD FACSAria. Single cells with CD3+, F4/80+ or Gr1+ (~10,000) were sorted into tubes containing 50 uL lysis buffer in the PicoPure™ RNA Isolation Kit (Thermo Fisher Scientific, KIT0204).

Quantitative PCR

Total RNA was isolated using PicoPure™ RNA Isolation Kit (Thermo Fisher Scientific, KIT0204) and reverse-transcribed using qScript cDNA Synthesis Kit (Quantabio, 95047-100) following the manufacture's protocol. Quantitative PCR was performed using 2x SYBR Green qPCR Master Mix (Bimake, B21202). Primers were synthesized by Integrated DNA Technologies and the sequences are listed in Supplementary Table 2. Gene expression level was calculated relative to β-actin using ΔCt values.
